# Interventions targeting sexual and reproductive health and rights outcomes of young people living with HIV: a comprehensive review of current interventions from sub-Saharan Africa

**DOI:** 10.3402/gha.v8.28454

**Published:** 2015-11-02

**Authors:** Leandri Pretorius, Andrew Gibbs, Tamaryn Crankshaw, Samantha Willan

**Affiliations:** Health Economics and HIV/AIDS Research Division (HEARD), University of KwaZulu-Natal (UKZN), Durban, South Africa

**Keywords:** intervention, young people, HIV, sub-Saharan Africa, sexual and reproductive health and rights

## Abstract

**Background:**

A growing number of young people (ages 10–24) are living with HIV (YPLWH) in sub-Saharan Africa (SSA). These YPLWH have particular needs and challenges related to their sexual and reproductive health and rights (SRHR). Contextual factors including gender inequalities, violence, stigma, and discrimination and lack of tailored services undermine YPLWH's SRHR.

**Objective:**

Understand the scope and impact of interventions targeting YPLWH to improve SRH-related outcomes in SSA.

**Design:**

We undertook a review to synthesise evaluated interventions (qualitative, quantitative, or mixed methods) aimed at improving the SRH outcomes of YPLWH in SSA with outcomes based on a World Health Organization framework of comprehensive SRHR approaches for women living with HIV. Using inclusion criteria, only six interventions were identified.

**Results:**

Interventions sought to improve a range of direct and indirect SRH outcomes, including sexual behaviour, adherence, disclosure, and mental health. Four overarching issues emerged: 1) all interventions were structured according to cognitive behavioural therapy theories of behaviour change – while showing promise they do not tackle the wider gender, social, and economic contexts that shape YPLWH's SRH; 2) ‘significant others’ were included in two of the interventions, but further work needs to consider how to leverage parental/guardian support appropriately; 3) interventions only accessed young people who were already linked to care, participants were likely to have better SRH outcomes than those potentially more vulnerable YPLWH; and 4) none of the interventions explored the sexuality of young people.

**Conclusions:**

There have been a limited number of evaluated interventions to strengthen SRH of YPLWH in SSA, and gaps exist in addressing the SRHR needs of YPLWH. Intervention approaches require greater scope and depth, including the need to address structural and contextual challenges.

Young people (ages 10–24) currently account for one-third of the total population in Eastern and Southern Africa (ESA) (1). As they reach puberty, they face new challenges related to their sexual and reproductive health and rights (SRHR) (2). Because of their emerging sexuality, young people undergo transformations of body image and identity, often at the same time that they become sexually active. Sexual activity also includes negotiating intimate relationships, managing one's fertility, and protecting against sexually transmitted infections (STIs) (3).

Evidence from sub-Saharan Africa (SSA) shows young people have poor SRHR outcomes (4), with high rates of unintended pregnancy, STIs, and limited condom use (5–7). Factors underlying these poor outcomes include gender inequalities – specifically the high rates of intimate partner violence (IPV) and harmful gender norms (5), – and poverty and economic marginalisation (8). More widely, denial and judgement about young people's sexuality limits their access to comprehensive knowledge, as well as the services and commodities used to protect and promote their sexual and reproductive health (SRH) (9, 10).

A key SRH issue for young people in SSA is the high levels of HIV-prevalence and incidence (7, 11). Globally two-thirds of all youth (ages 15–24) living with HIV live in this region (11), with an estimated 430,000 young people acquiring HIV every year (1). Furthermore, young women (ages 15–24) in ESA are two to four times more likely to be living with HIV compared to young men of this age (12).

The SRH needs and experiences of young people living with HIV (YPLWH) are considerably different to those not living with HIV (13, 14). YPLWH face the same challenges of dealing with their HIV status as adults living with HIV, such as disclosure of their HIV status, safer conception, safe sex, stigma, and treatment adherence (15–17), but additionally have to contend with the wider issues faced by all young people growing up. In addition, research suggests there may be differences in SRH experiences, challenges, and requirements between young people who acquired HIV vertically^1^ and have grown up living with HIV (18, 19), and young people who acquired HIV horizontally.

The World Health Organization (WHO) developed a conceptual framework outlining a comprehensive SRHR approach for women living with HIV (20). This is centred around five ‘pillars’ namely: sex life, pregnancy and fertility, treatment and its side effects, financial security, and mental health, all of which are identified as critical for ensuring a comprehensive approach to SRHR. We discuss these pillars in turn with a focus on how they relate to YPLWH, a focus which is lacking in much of the research and literature.

## Sex life

A comprehensive SRHR approach acknowledges that people, including young people, have the right to a pleasurable and satisfying sex life (21). WHO (20) recognises the particular challenges for women living with HIV in achieving a pleasurable sex life as well as negotiating condom use, especially in the context of gender inequalities and IPV (22). For YPLWH these issues are exacerbated, with the complexities of an emerging sexuality, which is often denied by adults, overlapping with the challenges of living with HIV.

WHO (20) strongly emphasised that women living with HIV who had a healthy sex life had disclosed their HIV status to their partner and their partner had accepted their HIV status. Moreover, studies suggest that disclosure is also closely linked to higher levels of social support and adherence to antiretroviral therapy (ART) (23) highlighting the importance of being able to disclose safely in intimate relationships.

In addition, women in SSA generally experience high levels of IPV (24), undermining their ability to achieve a satisfying sex life, including negotiating condom use (24, 25). For young women (ages 15–24) there is evidence that they experience higher incidence of IPV compared to older women (5). Moreover, there is an increasing body of evidence that shows that disclosure of HIV status by women to their partners can result in abandonment and violence (26).

## Treatment adherence and side effects

Medically, adherence to ART is required to decrease opportunistic infections and to ensure virological suppression (3). Improved overall health substantially affects overall well-being, including SRH (27). In addition, access and adherence to ART is also critical for safer conception (28). YPLWH experience numerous barriers to adherence, especially those who acquired HIV vertically (29). Studies suggest young people exhibit lower levels of adherence than adults (3) and have high loss to follow up while enrolled in treatment (30). A range of factors shape adherence, including social support, poverty, and health care quality (31).

The side effects experienced from taking necessary ART to prevent HIV progression is potentially a factor influencing the SRHR experiences of YPLWH (20). Evidence suggests that possible side effects of ART (and perhaps HIV infection itself) may negatively impact on sexual functioning – including decreased libido and erectile dysfunction – further affecting the SRHR of YPLWH (32).

## Fertility and family planning

The right to have children, to make informed choices about the number and spacing of children, and to prevent pregnancies if desired, is a central component of SRHR (33). Family planning services are very limited and unsupportive for all young people (34). YPLWH face additional stigma and discrimination from health care providers around their right to have children (35). Additionally, there exists a lack of knowledge among service providers about the specific contraceptive and safer conception needs of YPLWH (17, 16).

## Mental health

WHO (20) highlights the importance of good mental health to the overall SRHR of women living with HIV. Poor mental health is strongly related to negative health outcomes for young people, including poor SRH (36). YPLWH are more susceptible to depression and anxiety and other mental health issues compared to their peers who have not acquired HIV (37, 38). Increased social support has the potential to improve the mental health of YPLWH (39).

## Financial security

WHO (20) identifies a clear relationship between financial insecurity and poor SRHR outcomes. Financial insecurity places women in economically dependent relationships with men (40), limiting women's autonomy to make decisions, including around SRH (41). Moreover, women who are in economically dependent relationships lack negotiating power and are more likely to experience IPV (42, 43). High levels of poverty – particularly in contexts of gender inequalities – undermine women's mental health (20), which in turn undermines their SRHR.

**Fig. 1:**
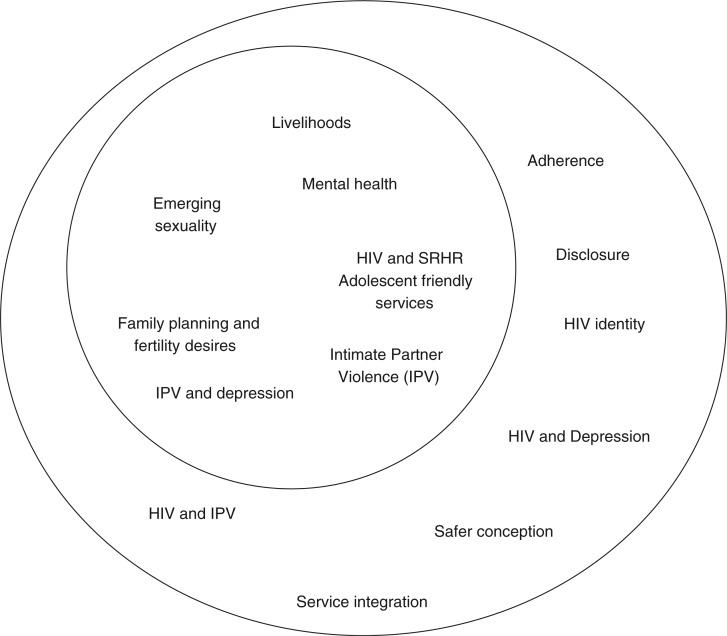
Sexual and reproductive health needs of young people and young people living with HIV.

Figure 1 captures the SRHR challenges experienced by all young people by means of the smaller circle and the additional challenges faced by YPLWH in the larger circle. This highlights the particular needs and requirements for SRHR of YPLWH and why specific services and interventions are required.

Given that YPLWH have unique and particular SRHR challenges, interventions have started to be developed and evaluated to support and promote their SRHR. In this article, we undertook a critical synthesis of current interventions that sought to strengthen the SRHR of YPLWH in SSA. We ask two questions: first, for YPLWH, what are the interventions that seek to promote their SRHR needs and experiences in SSA? Second, how are these interventions conceptualised and what lessons are there that might be useful for future SRH programming?

This review is a not a meta-analysis or a systematic review. First, there were insufficient studies with outcomes to make them directly comparable. Second, we are interested in conceptualising how the interventions were framed in theory and practice and who they targeted, rather than comparing study methodologies and outcomes.

## Methods

We conducted a review of interventions that sought to strengthen the SRHR outcomes of YPLWH (ages 10–24). Given the contested and unclear nature of what constitutes SRHR, we used the WHO's (20) five pillars (sex life, pregnancy and fertility, treatment and side effects, financial security, and mental health) to provide a framework for outcomes.

### Intervention review

**Fig. 2:**
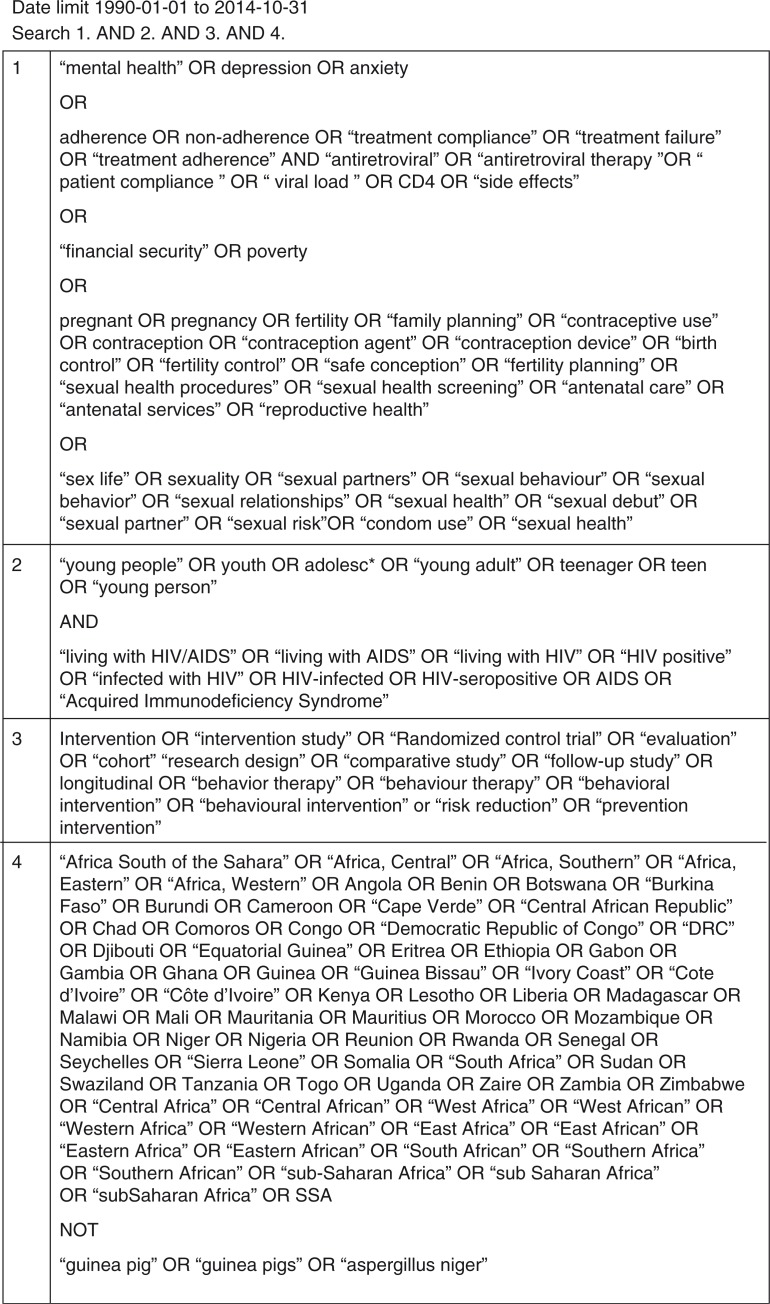
Search string.

We searched PsychInfo, Academic Search Complete, PubMed, and Google Scholar to identify published articles or grey literature. The terms searched thematically were: 1) SRHR outcomes – focused around the five pillars of WHO (20); 2) YPLWH; 3) Intervention – we included MeSH terms of these key words. Figure 2 shows the search equation that was used in this process of article identification. In addition, reference lists and bibliographies of relevant articles and reviews were also searched to identify additional articles.

Articles were included in this review if they were published between 1 January 1990 and 31 October 2014 and in English. In addition, the included interventions had to:

1. be evaluated based on qualitative, quantitative, or mixed data collection methods;
2. focus on young people (ages 10–24 years) or where the median age is in this age category, living with HIV;
3. be carried out in SSA; and
4. target and report on one of the predefined concepts of SRHR outcomes drawn from WHO (20).

Exclusion criteria include:

1. articles describing the SRHR needs and experiences of YPLWH,
2. interventions targeting all youth, not necessarily those living with HIV,
3. interventions dealing with adult or child populations,
4. interventions from geographical areas other than SSA, and
5. interventions targeting and reporting outcomes that might more widely affect SRHR of YPLWH, not stipulated in the WHO model.

**Fig. 3:**
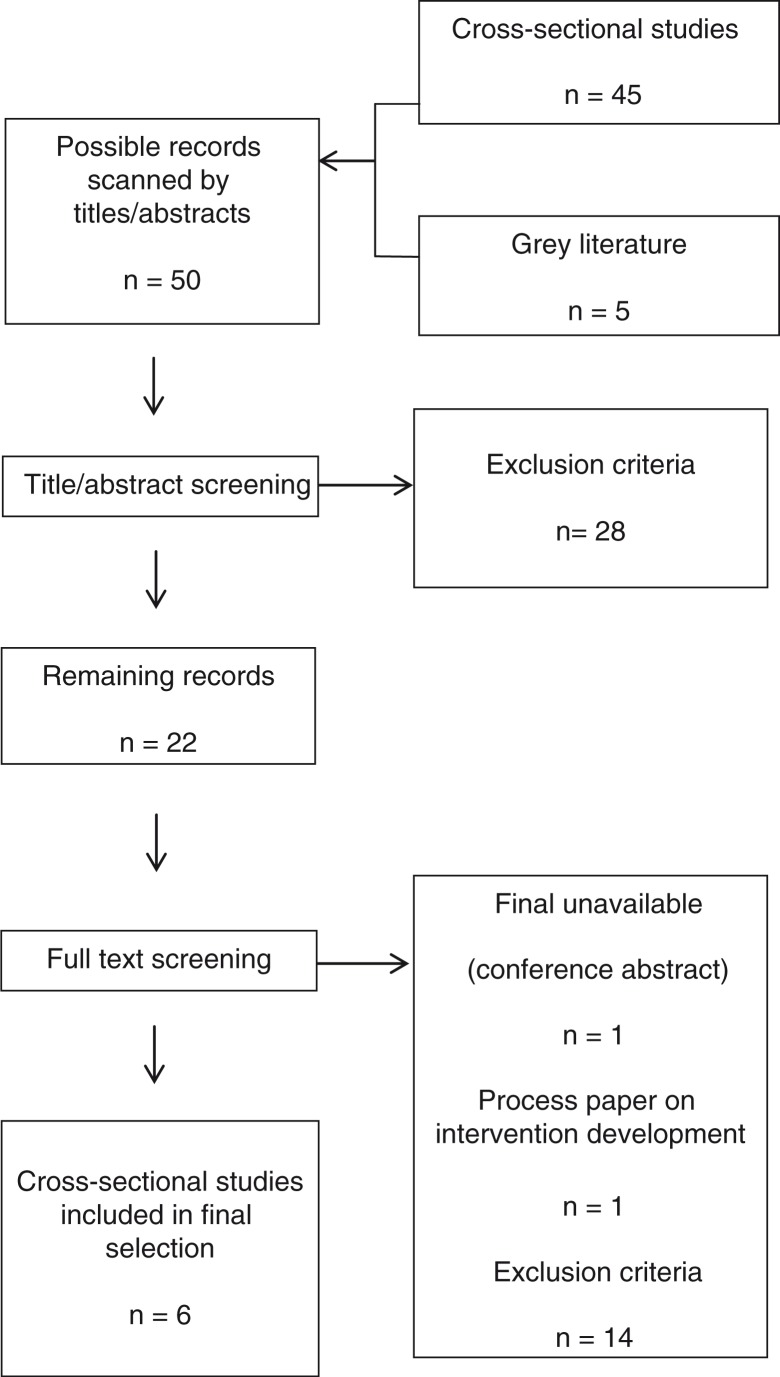
Flowchart of the process of study selection.

Articles were initially sorted by title and downloaded into reference management software, Endnote X7, before abstract screening. The abstracts were reviewed according to the inclusion criteria; if the inclusion or exclusion of an article was uncertain when reviewing the abstract, the full article was reviewed thereafter. Full text screening was conducted where this process refined the articles selected according to the same inclusion criteria. If there was any uncertainty about whether an article should be included in the study, a discussion between two authors took place. The full process is outlined in Fig. 3.

### Data extraction and analysis

Data were manually extracted from articles into two pre-developed data extraction tables based on the research questions asked of the data – the tables were populated by means of extracting the relevant information (Tables 1 and 2). Outcomes recorded in Table 2 were extracted around the WHO (20) conceptual framework.

**Table 1:** Intervention description of interventions for young people living with HIV

	Country	Theoretical approach	Intervention type and duration	Target group and transmission type (vertical/horizontal)
Mixed methods				
Snyder et al. (2014) (Hlanganani)	South Africa	Cognitive behavioural therapy (CBT), Bandura's social cognitive theory of self-efficacy	Support groups 3 sessions over 11 months 120 min per session	16–24 years Diagnosis up to 12 months prior Unknown
Bhana et al. (2014) (VUKA)	South Africa	Psychosocial support	Family-based programme with caregiver 10 sessions over 3 months	10–14 years Enrolled at HIV care at hospital Aware of his/her HIV status Vertically acquired HIV
Quantitative				
Lightfoot et al. (2007)	Uganda	CBT	One-on-one with nurses 18 sessions	14–21 years Unknown
Senyonyi et al. (2012)	Uganda	CBT	Group counselling Recurring weekly sessions 80 min per session	12–18 years Vertically acquired HIV
Qualitative				
Mupambireyi et al. (2014)	Zimbabwe	Psychosocial support	Support groups Meet once a week	11–13 years Knowledge of HIV status for at least 6 months prior Vertically acquired HIV
Parker et al. (2013)	DRC	Psychosocial support	Support group with trusted person 6 sessions over a period of 6 weeks 90 min per session	15–24 Aware of his/her HIV status Agree to Focus Group Discussions following session 2 and 6 Agree to attending the entire intervention Unknown

**Table 2:** Outcomes and results of interventions for young people living with HIV

	Study type, sample size, and intervention recruitment and retention	Follow-up time for evaluation	SRHR outcomes	Results
Mixed methods				
Snyder et al. (2014) (Hlanganani)	Randomised control trial (RCT) Intervention: 109 74 (68%) returning for all three sessions Control: 222 Male=5 (5%) Female=104 (95%)	Start of session 1 (baseline) to end of session 3 (follow-up)	1) Mental health	1) Improvement in attitude towards HIV as a manageable chronic disease – 9% increase, *p*=0.07. Increase in coping – 6.6% (*p*=0.2) declared increased feelings of support.
			2) Sex life	2) Disclosure: Improvement – six people disclosed during intervention with a mean number of people that participants disclosed to rose from 2 to 4. Condom use: In last 3 weeks, they had talked to their partners about condoms, rose 69 to 83% (*p=*0.035) and condom use increased by 12% (*p=*0.49).
			3) Treatment and side effects	3) ART attendance: Significantly higher proportion of intervention arm participants attended for their first ART visit (100%) vs. comparison arm, 58.06% (*p*<0.001, CI–0.66, −0.33).
				*Qualitative findings* Information on positive prevention and STIs were two of the most popular educational items: ‘About CD4 counts and ARVs – that you must know your viral load and CD4 count. After going to Hlanganani I went for a CD4 count test, and they found that I had a very low CD4 count, so they told me I should go on ARV treatment, so now I am starting treatment soon’. In learning about living positively with HIV, there was a trend towards significant improvement in managing HIV as a chronic disease. ‘Do people with HIV still live long and productive lives?’ There was a 9% increase in ‘Yes’ answers from intervention group (*p*=0.07).
Bhana et al. (2014) (VUKA)	RCT 74 families (caregiver plus child) enrolled in programme 65 participated in study Intervention: 33 Control: 32 59 (91%) completed post-test Females=33 Males=32	Two weeks after last intervention session	1) Treatment and side effects	1) Significantly greater improvements in reported adherence (*β*=1.5, *p*<0.05) There was also an improvement in treatment knowledge (*β*=0.27, *p*<0.08). *Qualitative findings* This article only reports qualitative findings for the caregivers of the young people.
Quantitative				
Lightfoot et al. (2007)	RCT Intervention: 50 Control: 50 Female=72% Male=28%	Assessed at baseline and 3 months	1) Sex Life	1) Intervention (34%) and control (46%) recently sexually active; remained similar in follow-up (35 and 44%). No change on number of sexual acts. Sexual partners: decrease in the log number of sexual partners (F1,19=4.68, *p*=0.04); Consistent condom use: Consistent condom use increased to 93% in intervention (*p*<0.01) from 10%, control did not significantly change, from 15 to 12%. Over half intervention and control highly protected (74 and 65%) (abstinence/consistent condom use), significantly rose to 98% intervention (*p*<0.01), no change in control.
Senyonyi et al. (2012)	RCT 328 contacted to participate via phone or face-to-face 171 were selected to take part in the intervention, 115 attended at least three sessions, completed one or more of assessments/instruments Female=54 (53%)	No information	1) Sex life	1) No significant group differences on sexual behaviour when compared to control (*p*=0.876).
			2) Mental health	2) Depression: No significant differences in depression variable (*p*=0.700). Anxiety: Mean scores in group showed significant decrease in anxiety (*p*=0.006).
Qualitative				
Mupambireyi et al. (2014)	Purposive sampling Part of a bigger clinical ART RCT Caregivers also interviewed Phase 1=26 Phase 2 and 3=15 Phase 4=12 Females=14 Males=12	At various stages of intervention (data collected over 15 months)	1) Treatment and side effects	1) Key message of support groups is to adhere for life, participants found it portrayed in a more understandable and in a helpful way than at clinics and home. ‘I would say you must attend support groups so that you are taught on taking medication well without missing because if you do not take medication well, you will die’. Still had adherence challenges, they felt that if they did not adhere, they were considered ‘bad’ and would not attend as a result. Children felt that the support groups were a good source of HIV information, thought when tested, they still had suboptimal knowledge of HIV and ARVs.
			2) Mental health	2) Easier to socialise and play, considered ‘normal’, ‘fit in’. Acceptance and self-acceptance, gained confidence, realised there were others like them. Felt role models/older children inspiring and transformative.
			3) Sex life	3) Deterred by support groups for fear of accidental disclosure to non-HIV peers. Forced disclosure as intro to group.
Parker et al. (2013)	Not specified 191 were eligible, part of programme FGD=13	FGD after session 2 and after session 6	1) Sex life	1) Improving condom use skills, improving ability to deal with unsafe sex, increasing capacity to assess level of risk of different behaviours. Improved ability to negotiate safer sex behaviour and openness to discuss sexual behaviour with caregiver.
			2) Mental health	2) Improving ability to deal with triggers of high-risk behaviour. Young people living with HIV reported improving problem-solving skills Increased decision-making autonomy, Improved ability to develop potential future goals.

Data were extracted by two members of the review team to ensure reliability of the review results. Following the extraction of data, a narrative analysis was performed on the data, whereby the differences and similarities across the articles were examined – this formed distinct topics for discussion.

## Results

We identified six interventions that met the inclusion criteria. Two interventions were in South Africa, two in Uganda, and one each in Zimbabwe and the Democratic Republic of Congo (DRC).

There was significant variation in intervention focus, as such interventions are not directly comparable. This is primarily due to the inclusive approach of the review. In the results, we focus first on intervention design and delivery and second on providing a narrative account of reported changes guided by the conceptual framework of WHO (20).

### Intervention design and delivery

All interventions used cognitive behavioural therapy (CBT), or variations thereof, as their theoretical model of intervention. CBT supports people to recognise potential ‘triggers’ of behaviours and then identify strategies to minimise triggers and develop behavioural alternatives to triggers. The Hlanganani intervention (44) used social cognitive theory in addition to CBT. Social cognitive theory builds on CBT, but also includes the concept of self-efficacy (45). Three interventions also used psychosocial support as a theoretical approach to change (46–48). Psychosocial support theories recognise that people are likely to have better health and improve their decisions if they feel supported by liked and trusted peers or family members (45).

All interventions were group-based in delivery, but few described whether these were separated by sex, age, or nature of HIV-acquisition, despite variations in SRH needs and experiences (18, 19). Parker et al. (48) used same sex groups, whereas Mupambireyi et al. (47) in Zimbabwe described dividing intervention groups across three distinct age categories (47). Three of the six interventions targeted youth who acquired HIV vertically (46, 47, 49), whereas it was unclear for the other interventions.

Two interventions also included ‘significant others’, as a way to build social support and ensure participants felt secure. Parker et al. (48) in the DRC describe participants bringing a trusted person, or ‘very important person’, to support groups (48). The VUKA programme in South Africa included a caregiver in support groups (46).

### Outcomes

We describe the outcomes of the interventions based on the WHO Framework (20) of Sex Life, Treatment and Side Effects, and Mental Health. No intervention addressed Financial Security or Fertility and Family Planning, despite these being critical components of a comprehensive SRHR approach.

#### Sex life

Five interventions reported a sexual behaviour outcome. Only one intervention, in Uganda, reported a significant change in sexual behaviour quantitatively (50), a decrease in the log number of sexual partners (*p=*0.04), and an increase in consistent condom use (*p<*0.01). They also showed a significant increase (*p<*0.01) in a combined measure of ‘highly protected’ defined as either abstinent or consistent condom use. Parker et al.'s (48) qualitative evaluation in the DRC reported improved condom use, ability to assess ‘levels of risk’, and negotiate safer sex, but there was no reflection on whether this differed by sex, given how safer sex is shaped by gender (48). Parker et al. also reported that participants were more open to discuss sexual behaviour with their caregiver (48).

The Hlanganani programme (44) reported a significant increase in participants talking to their partners about condoms in the past 3 weeks (*p=*0.035) by the end of the last session (follow-up). However, behavioural measures including condom use, although improving by 12%, were not significant (*p=*0.49). In Uganda, Senyonyi et al. (49) showed no significant group differences on sexual behaviour when compared to the control group, most likely because they focused on an abstinence-only approach (49), an approach for which there is no evidence of effect on behaviour change (51).

Only two interventions reported on participants’ disclosure to others. The Hlanganani programme (44) reported increasing disclosure by participants (44). In contrast, another intervention (47) in Zimbabwe reported participants felt reluctant to participate in support groups for fear of accidental disclosure to non-HIV peers. Additionally, the disclosure messaging conflicted with what participants were told at home – that they were to hide their status at all costs (47).

#### Mental health

Four studies reported mental health outcomes. In general, quantitative measures showed mixed outcomes for mental health, whereas qualitative data typically showed positive outcomes. The Hlanganani programme showed a small (6.6%, *p*=0.2) increase in participants feeling supported by others around their HIV status (44). Participants also reported improved attitudes towards HIV infection as a manageable chronic disease [a 9% increase (*p*=0.07)] (44). Senyonyi et al.'s intervention in Uganda with young people who vertically acquired HIV (49) showed no significant change in depression symptomology but did show a significant decrease in anxiety (*p*=0.006).

Qualitative findings were generally positive about the impact of interventions on the mental health of participants. In Zimbabwe (47), participants reported they gained peer acceptance, exhibited increased confidence and self-acceptance, and realised there were others ‘like them’. In addition, participants felt role models/older children in the group were inspiring. Participants in the DRC-based intervention reported improved problem-solving skills, increased decision-making autonomy, and the improved ability to develop potential future goals (48).

#### Treatment and side effects

Three interventions reported on treatment adherence. The VUKA programme (46) showed significant improvements in reported adherence (*p*<0.05). The Hlanganani programme (44) showed that a significantly higher proportion of participants attended for their first ART visit (100%) as opposed to the comparison arm (58.06%, *p*<0.001) (44). In the qualitative data from Zimbabwe, support group participants reported that adherence information was delivered in a more understandable and helpful way than at clinics and home (47).

## Discussion

We identified interventions seeking to improve the comprehensive SRHR of YPLWH in SSA, recognising that YPLWH face particular challenges in the realisation of their SRHR (14). In this discussion, we identify themes emerging across all interventions and reflect on how these interventions can be strengthened, specifically: 1) narrow theoretical backgrounds; 2) the inclusion of caregivers; 3) narrow recruitment strategies; 4) lack of discussion around the sexuality of young people.

### Dominance of CBT approaches

All interventions used CBT or variations thereof. Although CBT has been shown to have positive outcomes on a number of SRHR-related outcomes, including condom use (52) and mental health (53), it is considered to be narrowly individualistic and to ignore how wider social contexts shape human behaviour (54, 55). This is a particular challenge in SSA where poor SRHR outcomes for YPLWH are linked to broad structural factors such as high levels of poverty and gender inequalities, including IPV (56). CBT approaches, although demonstrating some positive change, fail to address these wider structural barriers to the realisation of YPLWH's SRHR.

In particular, no interventions focused on financial security of YPLWH, despite the clear links between financial security, adherence, and SRHR (20). More widely, poverty may undermine participation in interventions (57, 58); indeed in Zimbabwe, it was reported that financial insecurity undermined group participation (47).

In order to improve SRHR outcomes amongst YPLWH, a more effective approach may be to combine CBT strategies that seek to target ‘narrower’ more individualistic behaviours, with those focused on wider structural theories of health behaviour which seek to change gendered relationships, and reduce the drivers of IPV and financial insecurity.

### Including carers and ‘significant others’

Two interventions we identified included ‘significant others’, primarily caregivers. There is a strong rationale for the inclusion of caregivers in interventions; cross-sectional studies show caregiver involvement increases adherence in young people (39) and support by caregivers improves the psychosocial well-being of YPLWH (59). In addition, studies suggest caregiver support reduces sexual risk behaviours amongst young people (60). The incorporation of caregivers speaks to the wider concept of increasing the social support of YPLWH, and as such appears to be a promising approach.

There remains, however, a tension between engaging caregivers and supporting YPLWH's autonomy over their SRHR. Although social support has benefit to overall health, it may risk undermining YPLWH's right to privacy and autonomy in decision making about their lives. Young people's capacity to make informed decisions varies over time and is fraught with ethical considerations, which cannot simply be captured through either including or excluding significant others.

### Recruiting through clinics and health centres

An ongoing challenge for all interventions working with YPLWH is that they are likely to miss a substantial proportion of this population because they recruit through clinics and support centres. Although practically this makes sense in order to deliver an intervention in often challenging contexts, it is likely to exclude out-of-school youth and youth not engaged in care – population groups that are marginalised and more vulnerable to poor SRHR outcomes. In addition, interventions exclude young people lost along the treatment cascade. For young people the treatment cascade is steep, with many not beginning the treatment journey and more dropping out along the way (61). This further highlights the need for effective interventions to be developed and implemented, with innovative recruitment and retention strategies to ensure YPLWH remain on treatment and are part of wider interventions to improve SRHR.

### Sexuality

The interventions described in this article have a relatively narrow conceptualisation of sex and sexuality, in that they are focused on reducing sexual risk behaviour. Yet SRHR is a concept broader than sexual behaviour alone and includes issues such as fertility and effective contraception – issues not discussed in these interventions. Yet given the age of participants, it is likely that they may have questions about these topics. More widely SRHR needs to include the full spectrum of subjects such as sexuality and sexual identity, including issues of body image and sexual orientation (62, 63).

### Limitations

This review has the following limitations: It consulted a limited number of databases and sources of grey literature, possibly limiting the number of studies identified and included in the review. In addition, very few interventions were included in the analysis, limiting the generalisability of the review findings. It is likely that some interventions were excluded – mostly in grey literature – as they were not formally evaluated.

## Conclusions

This study undertook a critical review to identify and discuss interventions that seek to strengthen the SRHR of YPLWH in SSA. We found that there have been a limited number of interventions to improve SRHR outcomes of YPLWH in the region. At a very basic level, the number of evaluated interventions needs to increase, so that an evidence base can emerge around how to improve the SRHR of YPLWH.

In addition, our findings reflect that more detailed information regarding description of interventions, such as session content, sex and age, and delivery method, need to be provided so that others can more easily understand embedded theories of change and how interventions work. This information would advance understanding as to which components of the interventions worked best in contributing to positive outcomes (39). There also exists a need for broader, more inclusive interventions aimed at YPLWH that address structural and contextual issues, specifically gender equality and livelihoods strengthening, recognising how these are major determinants of realising SRHR for young people. More widely, understandings of SRHR remain narrow with limited engagement around sexuality and sexual identity. Only through articulating a comprehensive approach to improving SRHR that recognises the multiple underlying determinants, is it likely that the SRHR of YPLWH in SSA can be improved.

## Footnotes

1 This subgroup of young people living with HIV refers to those young people who acquired HIV *in utero* or at birth, by means of mother-to-child transmission. A second subgroup of young people living with HIV are often referred to as acquiring HIV ‘horizontally’ which refers to young people who acquired HIV through sexual transmission, injecting drug use, or blood transfusion.
